# Radiation and temperature drive diurnal variation of aerobic methane emissions from Scots pine canopy

**DOI:** 10.1073/pnas.2308516120

**Published:** 2023-12-21

**Authors:** Lukas Kohl, Salla A. M. Tenhovirta, Markku Koskinen, Anuliina Putkinen, Iikka Haikarainen, Tatu Polvinen, Luca Galeotti, Ivan Mammarella, Henri M. P. Siljanen, Thomas Matthew Robson, Bartosz Adamczyk, Mari Pihlatie

**Affiliations:** ^a^Department of Agricultural Sciences, Faculty of Agriculture and Forestry, University of Helsinki, Helsinki 00790, Finland; ^b^Institute for Atmosphere and Earth System Research/Forest Sciences, Faculty of Agriculture and Forestry, University of Helsinki, Helsinki 00790, Finland; ^c^Department of Environmental and Biological Sciences, University of Eastern Finland, Kuopio 70600, Finland; ^d^Department of Microbiology, Faculty of Agriculture and Forestry, University of Helsinki, Helsinki 00790, Finland; ^e^Institute for Atmosphere and Earth System Research/Physics, Faculty of Science, University of Helsinki, Helsinki 00560, Finland; ^f^Archaea Biology and Ecogenomics Unit, Department of Functional and Evolutionary Ecology, University of Vienna, Vienna 1030, Austria; ^g^National School of Forestry, University of Cumbria, Ambleside LA22 9BB, United Kingdom; ^h^Organismal and Evolutionary Biology (OEB), Faculty of Biological and Environmental Science, University of Helsinki, Helsinki 00790, Finland; ^i^Natural Resources Institute Finland (Luke), Helsinki 00790, Finland; ^j^Viikki Plant Science Center, University of Helsinki, Helsinki 00790, Finland

**Keywords:** aerobic methane production, diurnal cycle, Scots pine

## Abstract

Methane emissions from plant foliage may play an important role in the global methane cycle, but their size and the underlying source processes remain poorly understood. Here, we quantify methane fluxes from the shoots of Scots pine trees, a dominant tree species in boreal forests, to identify source processes and environmental drivers, and we evaluate whether these fluxes can be constrained at the ecosystem-level by eddy covariance flux measurements. We show that shoot-level measurements conducted in forest, garden, or greenhouse settings; on mature trees and saplings; manually and with an automated CO_2_-, temperature-, and water-controlled chamber system; and with multiple methane analyzers all resulted in comparable daytime fluxes (0.144 ± 0.019 to 0.375 ± 0.074 nmol CH_4_ g^−1^ foliar d.w. h^−1^). We further find that these emissions exhibit a pronounced diurnal cycle that closely follows photosynthetically active radiation and is further modulated by temperature. These diurnal patterns indicate that methane production is associated with diurnal cycle of sunlight, indicating that this production is either a byproduct of photosynthesis-associated biochemical reactions (e.g., the methionine cycle) or produced through nonenzymatic photochemical reactions in plant biomass. Moreover, we identified a light-dependent component in stand-level methane fluxes, which showed order-of-magnitude agreement with shoot-level measurements (0.968 ± 0.031 nmol CH_4_ g^−1^ h^−1^) and which provides an upper limit for shoot methane emissions.

## Significance

Reports of methane emissions from plant shoots led to a debate over the role of plants in the global methane cycle. Existing studies report strongly diverging results, likely due to differences in methodology and measurement artifacts, yet, few studies have quantified shoot methane fluxes under field conditions. Here, we measured the shoot methane exchange of boreal Scots pine trees from leaf-level laboratory to ecosystem-level field conditions. We find consistent and small methane emissions across scales; however, these emissions were orders of magnitude smaller than previously used emissions factors. We further show that shoot emissions exhibit clear diurnal trends that are driven by light and temperature.

Methane (CH_4_) is an important greenhouse gas responsible for one third of anthropogenic climate warming (1). Trees were recently recognized as an important component of the global methane cycle due to emission from their stems and shoots (2–5). These fluxes, however, have so far not been incorporated into global methane budgets due to uncertainties in estimates and poor mechanistic understanding (6, 7). Methane emissions from trees in boreal forests, and from tree shoots in general, have been rarely measured and remain particularly poorly constrained.

Trees emit methane that originates from three main source processes: i) the export of methane produced in anoxic soil layers through aerenchymas in gaseous form and dissolved in xylem water (8), ii) methanogenesis by endophytic archaea within plants, e.g. core rot; (9), and iii) aerobic methane production within plant tissues (10). Methane emissions from tree shoots are commonly attributed to aerobic methane production (11, 12), which remains by far the most cryptic and least understood of these processes. Such methane production in living plant foliage under aerobic conditions was first reported in ref. 10, followed by an intense debate as some studies succeeded in reproducing these results (13–15), while others failed to do so (16–18). Since then, isotope labelling has confirmed that methionine acts as a direct or indirect precursor of aerobically produced methane from plant tissues, thus proving that methane is produced from plant biomass (19). Further studies showed that plant methane emissions increase with UV irradiance (15) and visible light (20), temperature (21), and various stress conditions (13). Despite these efforts, the underlying biochemistry remains poorly understood and a subject of ongoing research (11, 22).

Due to this lack of mechanistic understanding as well as a scarcity of field measurements, the role of aerobic methane production in the global methane cycle remains poorly constrained. Global estimates of the emissions of methane derived from aerobic production in plant foliage range from 0 to 60 Tg CH_4_ y^−1^, accounting for up to 28% of all nonanthropogenic methane emissions (23–25). The wide range of these estimates and the large uncertainties within each estimate results from the limited number of field and laboratory measurements and from large uncertainties in upscaling these emissions. So far, no robust approach exists to quantify aerobically produced methane emissions at the ecosystem level, and leaf-level measurements under field conditions remain rare and contradictory (5, 26–30). Measurements of shoot methane exchange have been conducted with different combinations of chamber systems and analytical methods, most of which are still under development and prone to measurement artifacts and interferences (12, 31, 32). Global estimates of methane emissions from aerobic methane production in plants therefore still rely on emission factors derived from laboratory studies, and most estimates published so far (23–25) still rely on the emission factors initially published by ref. 10. These large uncertainties and the lack of field verification have thus far prevented the inclusion of aerobic methane production into global methane budgets even though many estimates exceed other processes mentioned in these budgets (2, 6).

One common way to partition ecosystem fluxes of trace gases is by analyzing the diurnal patterns of these fluxes and by comparing their temporal variation with environmental variables like solar radiation and temperature. Many plant-based processes are directly affected by light and therefore exhibit strong diurnal cycles, while soil-hosted processes are only indirectly affected by irradiation (e.g., through warming, drying, or plant root exudation) and show only weak diurnal variation. Studying the covariation of ecosystem methane fluxes with irradiation, e.g., during diurnal cycles, can therefore provide an important tool to quantify aerobically produced methane emissions at the ecosystem level, or at least provide an upper limit to such fluxes. A comparison of the diurnal patterns of shoot methane exchange with those of photosynthesis, transpiration, and stomatal conductance could further our mechanistic knowledge of production of methane in tree shoots. Such analysis, however, first requires a robust understanding of if and how aerobic methane production varies with light conditions and throughout the diurnal cycle.

However, surprisingly, the diurnal dynamics of methane exchange of plant foliage have not been studied thus far. This is likely due to the technical challenges associated with quantifying shoot-level methane fluxes. In the past, such measurements were conducted by repeated gas sampling from the headspace of enclosed shoots or whole plants components and quantitation by gas chromatography (5, 28) or more recently by circulating air between an enclosure chamber and a manually connected online methane analyzer (12, 26, 30). Such measurements are laborious, thus limiting replication and temporal resolution, and can cause substantial disturbance to the studied plant due to warming in the enclosed air volume, CO_2_ depletion, and transpired water condensing on chamber walls (33). Optical methane analyzers may also suffer from spectral interferences from water vapor (12) and coemitted volatile organic compounds (31).

**Fig. 1.**
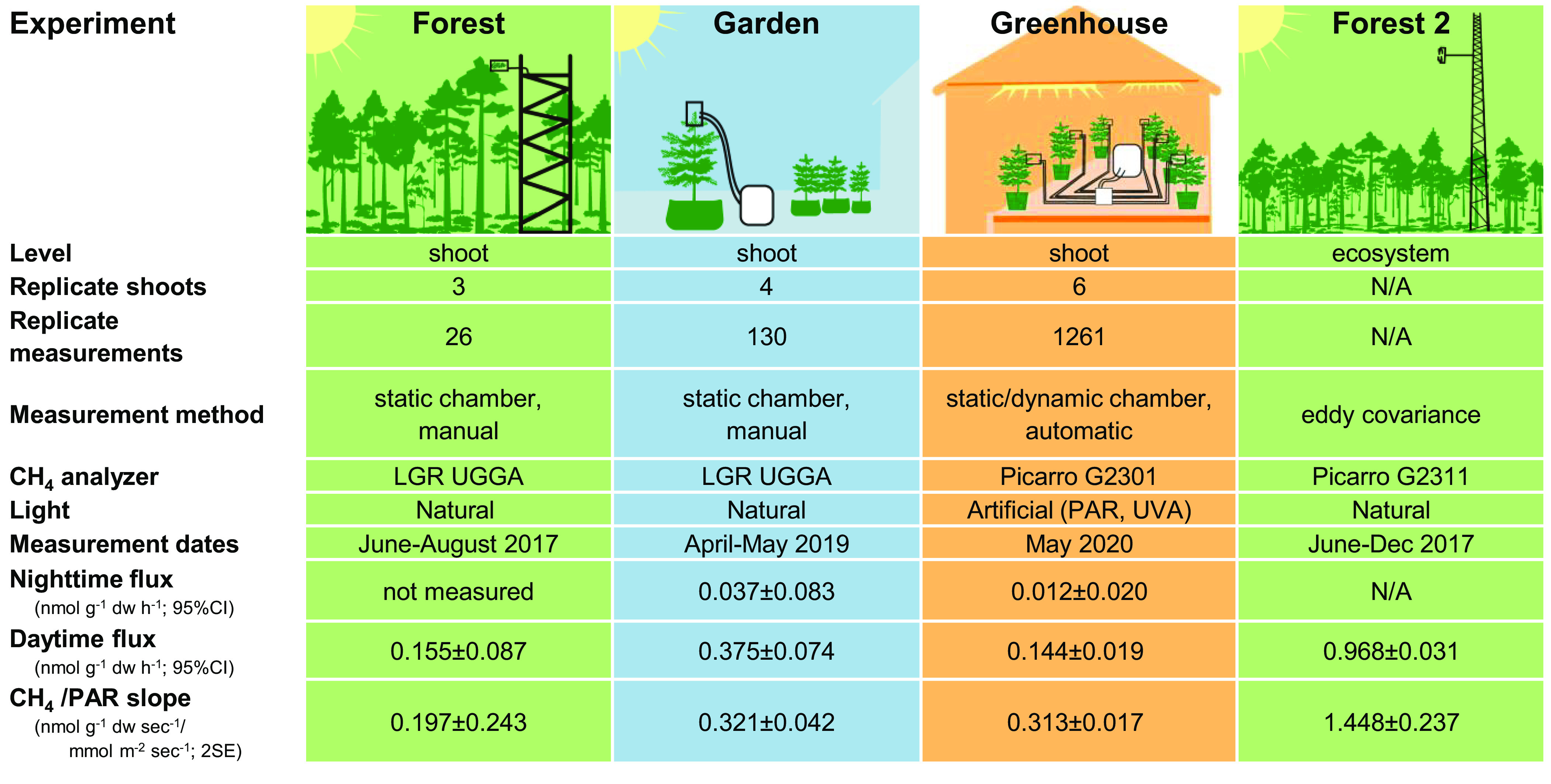
Overview of experiments and measurement results.

Here, we studied the diurnal patterns of shoot methane emissions using Scots pine (*Pinus sylvestris* L.), one of the most important boreal tree species, as a model plant. Our study was motivated by the initial findings of methane emissions from pine shoots in Hyytiälä Research Forest, a 55-y-old forest stand in southern Finland (5). In these initial measurements, however, methane exchange was quantified with manually operated shoot chambers that were shaded during the measurements to avoid excessive heating. They therefore provide little information about the light dependency and the diurnal patterns of methane fluxes. Here, we report the results from measurements conducted on mature trees in the field with unshaded chambers, followed by experiments with Scots pine saplings in garden and in greenhouse settings (Fig. 1). We then develop an approach to constrain aerobically derived methane emissions at the ecosystem level based on eddy covariance flux measurements and apply this approach to the Hyytiälä Research Forest. We discuss the relationship of the diurnal patterns of methane, CO_2_, and water fluxes, the drivers of methane emission dynamics, and its significance for determining the source process pathways.

## Results

### Scots Pine Shoots Emit Small Amounts of Methane.

**Fig. 2.**
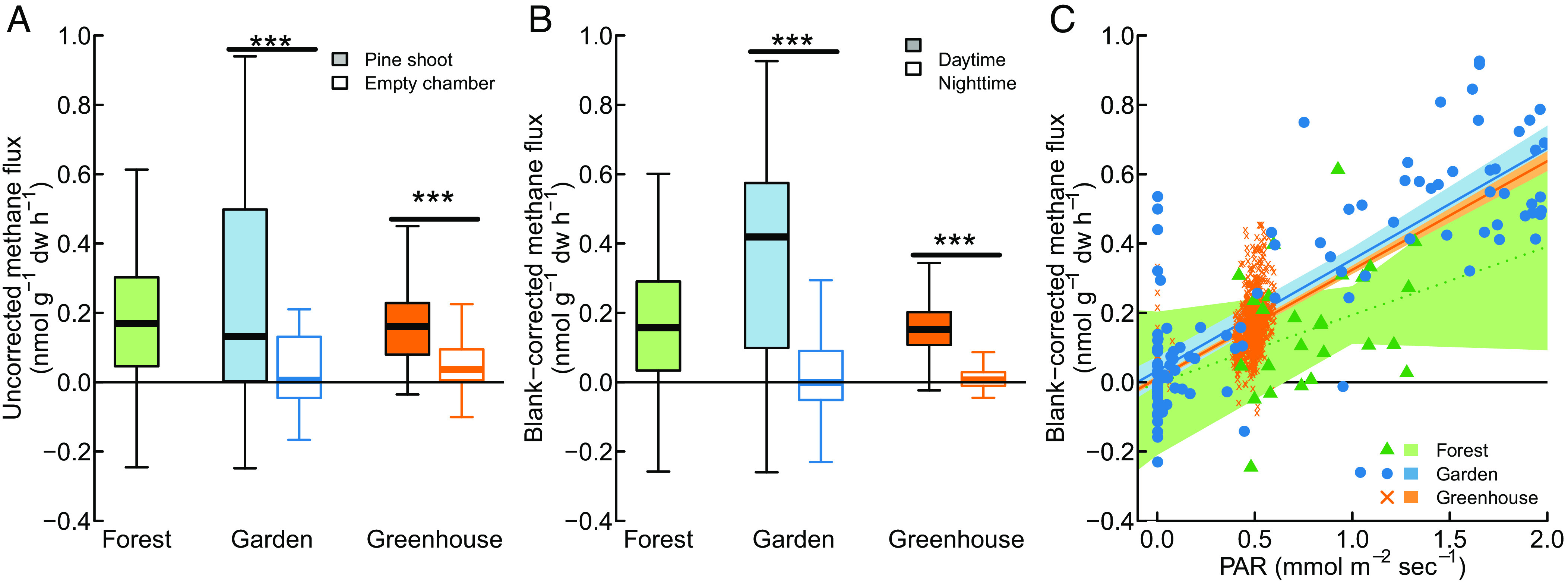
Methane fluxes from Scots pine shoots measured in forest, garden, and greenhouse settings; showing a comparison of measured methane fluxes and empty chamber controls (*A*); a comparison between daytime and nighttime fluxes (*B*); and between methane fluxes and photosynthetically active radiation (PAR) (*C*). Asterisks indicate significant differences (***P < 0.001).

In our initial measurements in the canopy of the Hyytiälä Research Forest in June–August 2017, conducted using manually connected cylindrical shoot chambers (*SI Appendix*, Fig. S1*A*) and a portable trace gas analyzer (LGR UGGA), we detected mean methane emissions of 0.167 ± 0.072 nmol g^−1^ foliar dry weight h^−1^ (Fig. 2*A*; *n* = 26; all results are reported as mean ± 95% CI). As shoot flux measurements are difficult to replicate under field conditions and canopy access in Hyytiälä was limited to three individual pine trees, we conducted further experiments with 3-y-old Scots pine saplings grown in the yard of the Viikki greenhouse facility of the University of Helsinki. As part of a larger study (12), we undertook two 24-h measurement campaigns in April and May 2019 where we conducted circa hourly measurements at the apical shoots of four Scots pine saplings using the same shoot chambers and greenhouse gas analyzer. Results were comparable to those measured in the Hyytiälä Forest canopy as shoots emitted 0.230 ± 0.049 nmol g^−1^ d.w. h^−1^ (*n* = 130). Empty chamber measurements with this system, conducted during the garden campaign, showed an apparent emission of 0.012 ± 0.049 nmol g^−1^ d.w. h^−1^ (*n* = 29; scaled to the average shoot foliar dry weight). After correcting for these empty chamber fluxes, we found that pine shoots emitted 0.155 ± 0.087 and 0.217 ± 0.069 nmol g^−1^ d.w. h^−1^ in the forest and garden campaigns, respectively.

To further increase the replication and temporal resolution of our measurements and to test whether our results were robust with regards to common artefacts of the static chamber method, we conducted experiments with a recently developed automated chamber system (33) (*SI Appendix*, Fig. S1*B*). This system uses a different trace gas analyzer (Picarro G2301), cools enclosure chambers to within 2 °C of ambient temperature, removes excess water through a membrane dryer, replaces the removed CO_2_ though automatic injections, and can measure CO_2_ and H_2_O exchange in a separate dynamic chamber mode. We used this system to measure methane fluxes at lateral shoots of six 2-y-old Scots pine saplings over a period of 33 d in May to June 2020. Again, we detected methane fluxes comparable to our forest and garden measurements: Measured methane emissions from pine shoots were 0.165 ± 0.006 nmol g^−1^ d.w. h^−1^ (*n* = 1,261), apparent fluxes in an empty chamber were 0.075 ± 0.034 nmol g^−1^ d.w. h^−1^ (*n* = 288), and empty-chamber-corrected methane emission rates were 0.090 ± 0.034 nmol g^−1^ d.w. h^−1^ (Fig. 2*A*).

### Shoot Methane Emissions Increase with Irradiation.

To assess whether shoot methane fluxes change throughout the diurnal cycle, we first tested whether fluxes during light periods (PAR > 0.05 mmol m^−2^ s^−1^) differed from those during dark periods (Fig. 2*B*). In the garden experiment, we found that daytime fluxes (0.375 ± 0.074 nmol g^−1^ d.w. h^−1^) were significantly larger than the average nighttime fluxes (0.037 ± 0.083 nmol g^−1^ d.w. h^−1^; *T* = 7.56, *P* < 0.001) and that nighttime fluxes were not significantly different from empty chamber measurements. The greenhouse experiment gave similar results with average fluxes of 0.144 ± 0.019 nmol g^−1^ d.w. h^−1^ during light periods and 0.012 ± 0.020 nmol g^−1^ d.w. h^−1^ during dark periods (*T* = 38.9, *P* < 0.001). Significant differences between daytime and nighttime fluxes were found for each individual shoot (*SI Appendix*, Fig. S2). We did not conduct night-time measurements in the forest campaign, but daytime fluxes in the forest (0.155 ± 0.087 nmol g^−1^ d.w. h^−1^) were comparable to the greenhouse experiment.

Both garden and greenhouse measurements showed that shoot methane emissions increased with incoming light, measured as PAR (Fig. 2*C*). This was best evidenced in the garden experiment, where we found a linear correlation between PAR and methane flux (*r* = 0.82, *P* < 0.001). The greenhouse measurements also showed such a correlation (*r* = 0.72, *P* < 0.001), although the data were bimodally distributed between light and dark periods. In the forest measurements, we found greater emissions at higher PAR, but this trend was not statistically significant (*r* = 0.31,*P* = 0.118), likely due to the lower number of replicate measurements and lower precision of field measurements. Nevertheless, measurements in all three settings showed in a similar CH_4_ flux: PAR slopes (0.197 to 0.322 nmol g^−1^ dw s^−1^ (mmol m^−2^ s^−1^)^−1^; Fig. 1)) and intercepts close to 0, indicating that methane fluxes from Scots pine shoots increase linearly with light intensity in both saplings and mature trees.

### Shoot Methane Emissions Follow Pronounced Diurnal Cycles.

**Fig. 3.**
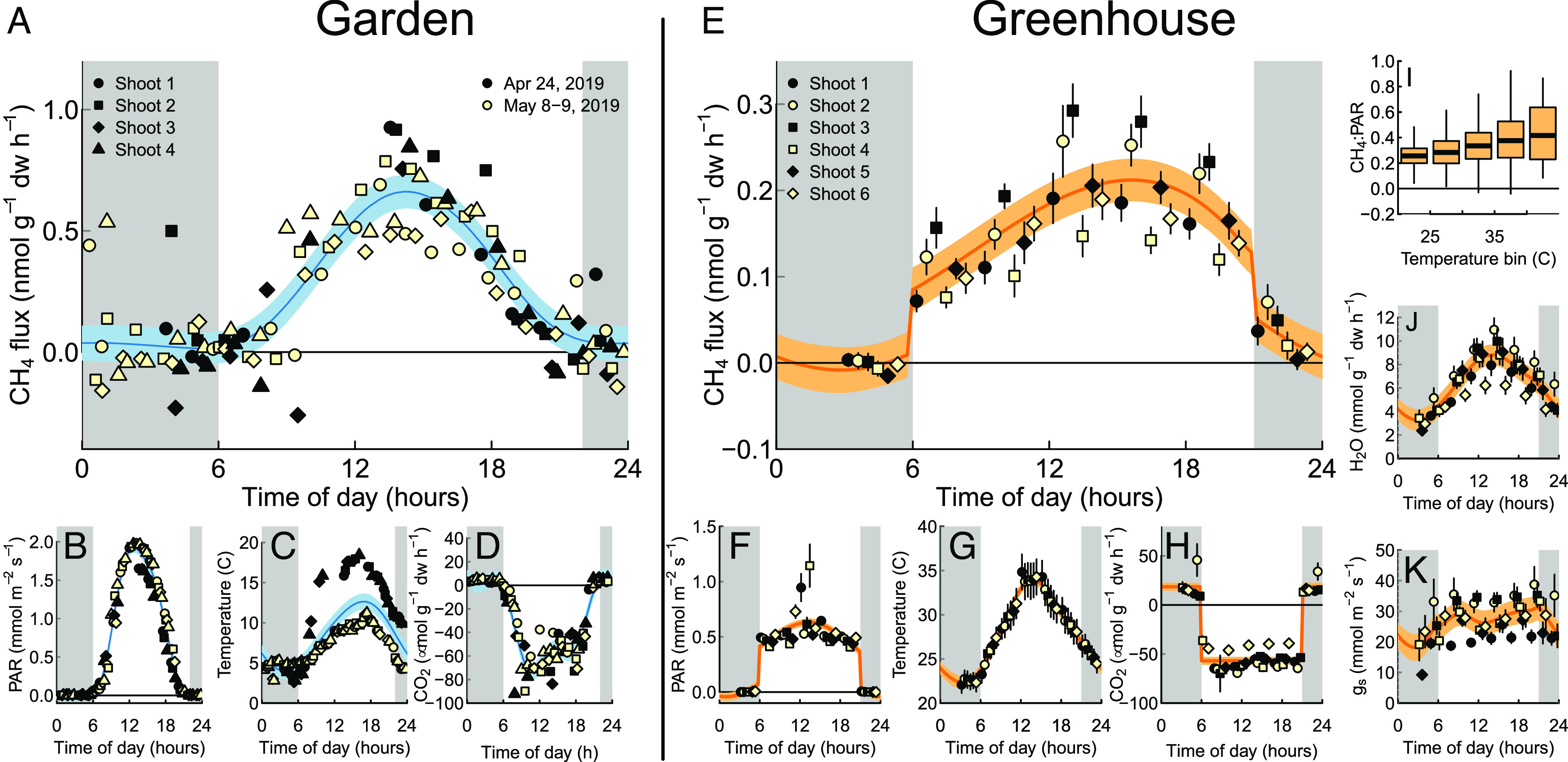
Diurnal trends of shoot methane fluxes measured on Scots pine saplings and other parameters (PAR, temperature, CO_2_ and water exchange, stomatal conductance) in garden (*A*–*D*) and greenhouse (*E*–*K*) experiments. Points indicate individual measurements (chamber closures) in *A*–*D*, and the mean of 33 measurements (one per day) in *E*–*K*. Error bars in *E*–*K* indicate 95% CIs (2 SEs). Purple lines represent a smoothing function (34) that accounts for random effects (individual shoot). Finally, shoot fluxes binned by chamber temperature (*I*), where bold lines indicate the median of each bin, boxes the interquartile range, and whiskers the estimated 95% range. PAR, photosynthetically active radiation; CO_2_, leaf CO_2_ exchange; CH_4_:PAR, methane emission (in pmol m^−2^ leaf area h^−1^) per unit PAR (mmol m^−2^ s^−1^); H_2_O, leaf water exchange, g_s_, stomatal conductance.

Following this initial analysis, we conducted a more detailed inspection of the diurnal patterns of methane fluxes along with environmental and gas exchange measures. In the garden experiment, we found that methane emission followed a bell-shaped curve during daytime, while remaining close to zero throughout nighttime (Fig. 3*A* and *SI Appendix*, Fig. S3). This pattern was similar to those of PAR and temperature (Fig. 3 *B* and *C*). CO_2_ uptake, in contrast, leveled off during mid-day while following similar trends as methane emissions during nighttime, morning, and afternoons (Fig. 3*D*).

Methane fluxes in the greenhouse experiment, in contrast, followed a box-shaped pattern with emissions during light periods and no fluxes during nighttime (Fig. 3*E* and *SI Appendix*, Fig. S4). This pattern was similar to PAR as the light sources were turned on and off (Fig. 3*F*), although methane fluxes showed a maximum during mid-afternoon not present for PAR. We also observed high PAR during individual mid-day measurements from spots of sunlight on individual shoots chambers but these were not matched by elevated methane emissions. Temperature, in contrast, followed a triangular pattern from ca 25 °C during nighttime 35 °C during mid-day (Fig. 3*G*). CO_2_ uptake (Fig. 3*H*) followed the pattern of PAR and did not show the mid-afternoon maximum observed in methane fluxes. The greenhouse experiment provided an opportunity to study the role of temperature in methane emissions as PAR varied little during daytime due to artificial lighting, whereas temperature varied within and between days. We therefore binned closures by temperature quintiles and compared the ratio CH_4_:PAR (Fig. 3*I*). The result demonstrated that CH4 fluxes at a given PAR level increased with temperature, similar to trends observed in the garden experiment (12).

Additional measurements in the greenhouse experiment also allow us to compare methane emission to water exchange and stomatal conductance. Transpiration fluxes showed diurnal trends that were distinct from CO_2_, methane, and PAR but similar to temperature (Fig. 3*J*). Transpiration decreased only partially during dark hours, from a maximum of 10 mmol g^−1^ d.w. h^−1^ to a minimum of 4 mmol g^−1^ h^−1^. Similarly, stomatal conductance was ca. 30% diminished, but not eliminated, during nighttime (Fig. 3*K*).

### Ecosystem-Level Estimates of Aerobic Methane Production.

**Fig. 4.**
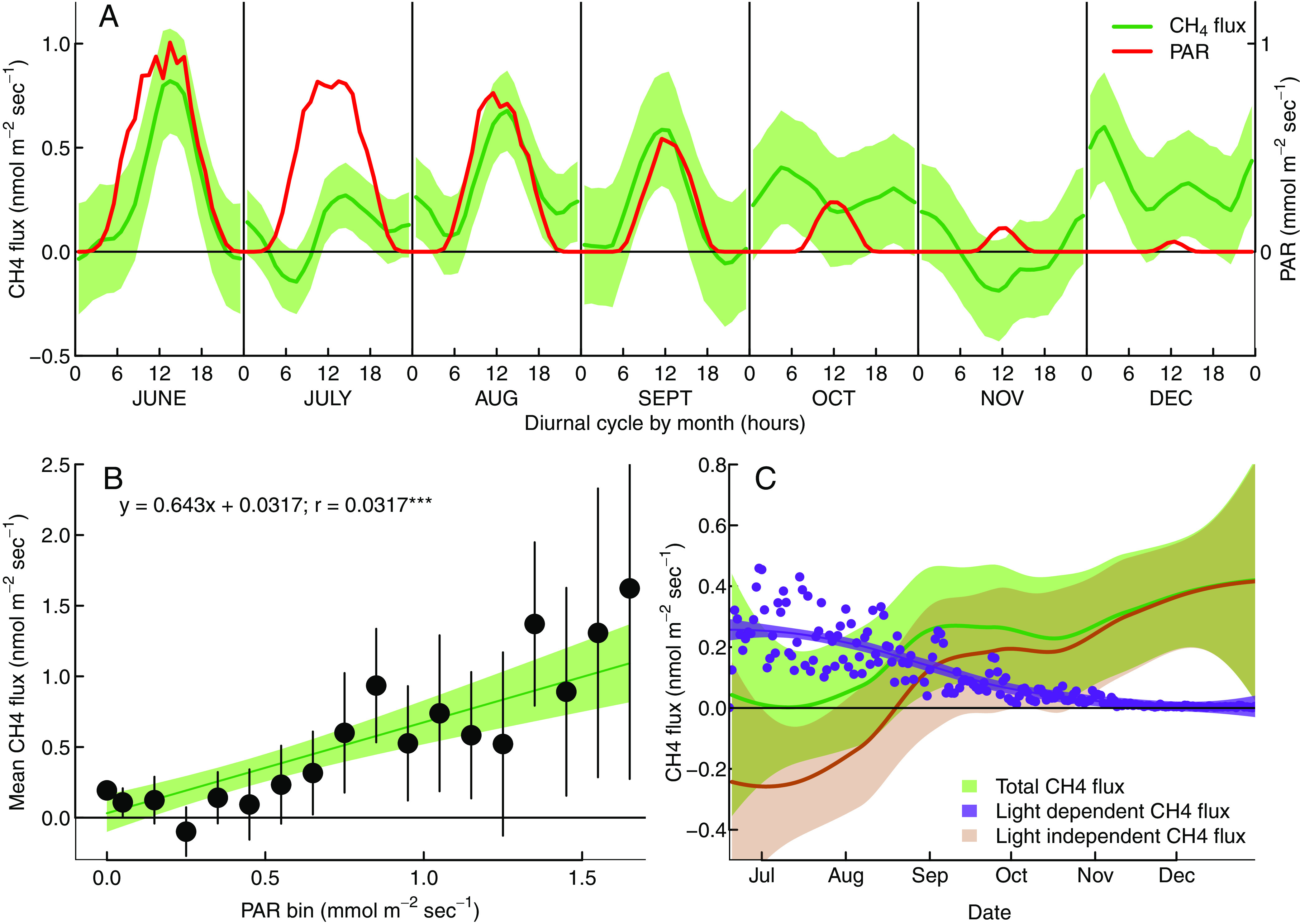
Monthly diurnal trends of PAR (yellow) and stand-level methane fluxes (purple) measured by eddy covariance (*A*). Further, correlation between irradiation photosynthetically active radiation (PAR) and ecosystem-level methane flux (measured by eddy covariance) at the Hyytiälä research forest after binning by PAR and averaging methane fluxes (*B*). Further, partitioning of the ecosystem-atmosphere into light-dependent and light-independent components (*C*), error bars and shaded areas indicate 95% CIs.

Based on our finding that shoot methane emissions follow light-dependent diurnal cycles, we analyzed whether such diurnal cycles can also be identified in micrometeorologically (eddy covariance) measured ecosystem-atmosphere methane fluxes. For this, we used a dataset of methane fluxes measured at the Hyytiälä Research Forest between June 15 and December 31 2017. A preliminary analysis (Fig. 4*A*) showed distinct diurnal patterns that paralleled PAR irradiation during most summer (June, August, September), although these trends were noteworthy absent during July. In contrast, little diurnal variation was found during October to December, when PAR intensity was low throughout the day. We tested whether the methane fluxes in this dataset were correlated to PAR and found a small (*R* = 0.032) but significant (*P* = 0.012) correlation (*SI Appendix*, Fig. S5). To further reduce the measurement noise, we binned the mean methane fluxes by PAR level and again found greater CH_4_ emissions at higher PAR (*R* = 0.881, *P* < 0.001, Fig. 4*B*). The slope between PAR and methane flux was 0.64 ± 0.10 nmol CH_4_ g m^−2^ (ground) s^−1^ (mmol m^−2^ s^−1^)^−1^.

We used this relationship to partition ecosystem-scale methane fluxes into a light-dependent and a light-independent component (Fig. 4*C*), assuming that the light-dependent component resulted from aerobic methane production. We find that this light-dependent flux fraction accounts for 0.0 to +0.4 nmol m^−2^ (ground) s^−1^, with a decreasing trend from summer to fall and winter (Fig. 4*C*). When scaled to foliar dry weight, this component accounts for 0.968 ± 0.031 nmol CH_4_ g^−1^ dw h^−1^ during July to December daytime hours. This top-down emission estimate is thus 3 to 8-fold larger than daytime fluxes measured at the leaf-level. In addition, shoot-level measurements were conducted during spring and summer months, when light levels are higher than in the July to December average, further increasing the divergence between top-down and bottom-up results. This divergence may have resulted from other light-dependent processes besides methane formation in foliage contributing to ecosystem-level methane fluxes. The light-dependent component of the ecosystem methane exchange should therefore be seen as the upper limit of aerobic methane production in these forests. Nevertheless, despite these significant differences, the order-of-magnitude agreement between top-down and bottom-up estimates is remarkable and gives further reliability to our result.

## Discussion

### Scots Pine Shoots Emit Small Amounts of Methane.

We find consistent evidence for methane emissions from Scots pine shoots in both adult trees and saplings, in forest, garden, and greenhouse settings, in automated and manual measurements, and with two methane analyzers from distinct manufacturers (Fig. 2). Moreover, we find order-of-magnitude agreement between chamber-based shoot-level and micrometeorological ecosystem-level flux measurements (Fig. 1). The difference between shoot and ecosystem-level measurements may have resulted from inaccuracies in measurements close to the detection limit, inaccuracies in the upscaling model, or from other methane emissions responsive to the diurnal cycle of plants, e.g., stem emissions (35). While these results were limited to a single species (Scots pine), the consistency results across diverse methods, settings, and specimens provide robust evidence for shoot methane emissions in this model system.

So far, only six studies quantified shoot methane exchange in field or field-like conditions (*SI Appendix*, Table S1), of which two report that tree foliage acts as a strong methane sinks (26, 30), one found that shoots were methane-neutral (27), and three found small emissions (5, 12, 28). Laboratory-based results also vary widely with emissions ranging from <0.01 to 23 nmol g^−1^ h^−1^. Our results are inconsistent with prior field studies showing strong methane uptake (26, 30), which clearly did not occur in any of our measurements. These different findings may have resulted form the distinct location, time, or species included in the different studies, but also from artifacts produced by the distinct measurement methods. We have previously shown, for example, that over time, the humidity correction applied by methane analyzers can drift away from its calibration value (12), which leaves the analyzers prone to interference from transpired water. This can cause measurement artefacts that appear like strong methane uptake. Similarly, a lack of methane emissions reported by both field and laboratory studies may have resulted from the use of UV-opaque chambers, e.g., ref. 27, thus potentially preventing photochemical reactions that cause methane production or from analytical methods that were unable to detect small methane fluxes as found in our study. In fact, our results fall well within the uncertainty (i.e., below the detection limit) of studies that report the absence of methane emissions (16–18) but are clearly inconsistent with the strong methane emissions reported in initial laboratory measurements (e.g. ref. 10). Our data thus indicate that while methane emissions are present in Scots pine shoots, these occur at rates much lower than reported in initial experiments, while prior studies reporting an absence of methane emissions likely did so because they lacked the sensitivity and replication to reliably detect these fluxes.

Our data include measurements that show apparent methane uptake by Scots pine shoots. Such uptake has been reported for tree shoots (29, 30) and stems (36, 37) and can result from the activity of methanotroph microorganisms in plants (29, 38). We cannot exclude that such methane uptake occurred in our study, but we did not detect conditions that showed consistent methane uptake, and the individual measurements showing apparent methane uptake did not exceed the measurement uncertainty.

### Methane Emissions Originated from Aerobic Methane Production.

We conducted multiple measurements and theoretical calculations to confirm that the measured shoot methane emissions resulted from aerobic methane production rather than other source processes, i.e., transport of soil CH_4_ via tree stems to the shoots or microbial methanogenesis in the foliage. Soil methane is often reported as the source of methane emitted from tree stems (e.g. refs. 8 and 39). Trees might transport soil-produced methane through diffusion in aerenchymes (39) and via advective transport of dissolved CH_4_ with xylem sap (40). Ref. 41 recently calculated how far soil methane can be transported in transpiration stream of Scots pine trees. They concluded that a typical mature tree cannot transport methane from the soil in transpiration stream up to the height of the canopy (10 to 15 m) and that most of the soil-derived methane is emitted through radial diffusion and emission through the bark. With saplings, the required distance of transport is much smaller and the transport of soil-derived methane to shoots hence could theoretically occur. To assess this possibility, we compared the amount of water transpired from Scots pine shoots to the measured aerobic methane emissions from the shoots and then estimated the concentration of methane in the soil needed to create the emission rate. The measured transpiration of Scots pine shoots remained <10 mmol g^−1^ h^−1^ (Fig. 3*J*). In order for this water to carry 0.2 nmol CH_4_ g^−1^ h^−1^ to the shoot, it would need to contain 1.11 μmol CH_4_ kg^−1^ H_2_O. This is theoretically possible as the solubility limit of methane in water is ~1.43 mmol kg^−1^ at 20 °C. Generating these methane concentrations, however, requires a methane concentration in the rooting zone equivalent 776 ppm in air (78 Pa partial pressure). This exceeds atmospheric methane concentration ∼ 300-fold and is unrealistic for upland soils both in our greenhouse soil pots and in upland forest soil. In the Hyytiälä research forest soil methane concentrations are typically below atmospheric levels (42), and methane concentrations in the stems mature pine tree were <10 ppm (*SI Appendix*, Fig. S6 and Table S2). Furthermore, methane and water fluxes from the shoots followed different diurnal cycles (Fig. 3), with methane emissions decreasing below the detection limit during nighttime even though transpiration continued, although at a lower level than during daytime.

Methane emitted by trees may also originate from microbial methane production, which can occur in the stems of some tree species (mainly Populus sp.) in particular during core rot. This, however, has only been reported for adult trees (9, 43, 44) and not at all for Scots pine trees. It is unlikely that such anaerobic microenvironments have formed in the stems of Scots pine saplings with a stem diameter <2 cm. Recent work also identified the presence of methane-producing microorganisms (Methanoregula and Methanothrix) in the conifer tree foliage (29), which could in theory contribute to the methane emissions from the foliage. To exclude this possibility, we analyzed the presence of methanogenesis-related functional genes (mcrA) in the Scots pine shoots of garden experiment through targeted metagenomics (see method in ref. 29), but we found no evidence for the presence of methanogens in our samples (*SI Appendix*, Table S3). Based on this, we can confidently say that methane-producing microbes play a minor role in the methane emissions from the studied Scots pine shoots.

### Implications for the Biochemistry of Aerobic Methane Production.

We find that Scots pine shoots emit methane with diurnal patterns that are similar to those of irradiation and photosynthesis but distinct from temperature, transpiration, or stomatal conductance. These results indicate that methane production in foliage results either from a direct photochemical reaction between light and biomass (15). We cannot rule out that methane is produced as a metabolic side product, e.g., in the methionine cycle (19, 45), if such metabolisms are linked of the diurnal cycle of plant C fixation. Distinguishing these potential source processes is difficult given that light and photosynthesis-dependent metabolisms covary in time in most natural and experimental settings. Future experiments to unravel the biochemistry underlying aerobic methane emissions should therefore aim at discriminating whether methane production driven by light or metabolic activity.

The abrupt end of methane emissions after lights were turned off (Fig. 3*E*) indicates that methane is not produced during nighttime and that concentrations inside the Scots pine needles do not build up during dark periods when stomatal conductance is lowered. This is also supported by the absence of a pulse of methane emissions due to stomata opening after lights are turned on (Fig. 3*E*), and unimpeded methane emissions (Fig. 3*A*) during midday when CO_2_ uptake is limited (Fig. 3*D*). This is consistent with findings of other highly volatile compounds (e.g., isoprene) whereas stomatal conductance limits the emissions of water-soluble organic compounds (e.g., methanol) (46). Our experiments can thus not distinguish whether methane was formed at the surface or the interior of the pine needles.

### Implications for the Incorporation of Aerobic Methane Production into Global Methane Budgets.

While the shoot and ecosystem level flux measurements conducted in this study show robust evidence for methane emissions, they also indicate that these emissions are two orders of magnitudes lower than the emission factors used in previous upscaling efforts (*SI Appendix*, Table S4). Most global estimates so far (10, 23–25) are based on the emission factor initially reported by ref. 10 (23.4 nmol g^−1^ dw h^−1^), resulting in estimates between from 8 and 149 Tg aerobic methane production per year (*SI Appendix*, Table S3). When the average emission factor measured in our study (0.155 nmol g^−1^ dw h^−1^, from forest measurements) is applied, we estimate a global emission of 0.05 to 0.99 Tg CH_4_ y^−1^ using the same upscaling models. This lower estimate is comparable to estimates based on independently measured emission factors of pectin under UV radiation (0.2 to 1 Tg CH_4_ y^−1^) (47). Similarly, past estimates of the annual aerobic methane production in boreal forests range 0.3 to 3.6 Tg CH_4_ y^−1^ based on the emission factor from ref. 10, and 0.0015 to 0.019 Tg y^−1^ based on the emission factor measured in our study. Our results thus show how initial, high estimates can be reconciled with later independent estimates if shoot-level emission factors are corrected downward based on field measurements.

We caution that limited data underpin our results. Our data are limited to systematic measurements of one species (Scots pine). We have also conducted sporadic measurements of two other boreal species (Norway spruce, silver birch; *SI Appendix*, Table S4), and if high fluxes (emission or uptake) were present in these species, we certainly would have detected them (28). Our estimates are thus reliable for European boreal forests. Beyond that, more field measurements, ideally both at the shoot and the ecosystem level and using a variety of methane analyzers to identify potential instrument biases, need to be conducted for a more robust evaluation of global estimates of shoot methane emissions and aerobic methane production. Nevertheless, we demonstrate that such reliable flux measurements are possible, even in the boreal biome where shoot methane emissions are comparatively low.

## Materials and Methods

### Field Sites.

The SMEAR II measurement station at Hyytiälä is located in a Scots pine dominate forest stand in southern Finland (61°51’ N, 24 °17’ E, 181 m altitude). The site is located on a haplic podsol soil over silty glacial till parent material. The forest was regenerated in 1962 by seeding after clear-felling and prescribed burning and is managed to mimic a local production forest. At the time of measurement (2017), trees had reached a height of 18 to 20 m. The forest was last thinned in 2002. Measurements were conducted on top of a scaffold tower located in a hilltop position. A detailed description of the forest stand is available in ref. 48. Shoot flux measurements were conducted weekly between July 7 and August 15 2017 on two Scots pine trees next to a scaffold tower.

### Garden and Greenhouse Growth Conditions.

Scots pine saplings for the garden experiment were obtained from a commercial grower (Huutokoski Ltd) in November 2017. The saplings were planted in 20-l pots using soil collected at the Hyytiälä research forest and buried in a sand bed in the garden at the Viikki greenhouse complex at University of Helsinki. By the time of our measurements the saplings had reached a height of 80 to 100 cm. We conducted two 24-h campaigns on two mostly cloud-free days (April 24 and May 8, 2019) using the same chamber technique as in the field. An empty chamber was placed in the immediate vicinity of the saplings and included into every measurement round.

Saplings for the greenhouse experiment were obtained from Harviala Ltd in Fall 2020. The saplings were potted in 15-L pots with peat soil and overwintered in the garden setting described above. In March 2020, 21 d before the start of the experiment, the saplings were transferred into a greenhouse compartment. The plants were illuminated for 15 h per day by LED lamps (type B100/ AP67, Valoya Oy, Helsinki, Finland) placed ca. 15 cm above the measured shoots. The trees were watered 1 L per pot weekly. Since these lamps did not emit the UV radiation of solar radiation present in outdoor environments, we supplemented the irradiance with UV-A/B lamps (QUV(C) fluorescent tubes, type UVA-340, Q-lab Corporation) placed ca. 20 cm above the shoots. The UV lamps were covered with a cellulose diacetate film, which attenuated any UV-C radiation emitted by the lamps. A spectrum of the irradiation arriving at the shoot chamber is provided in *SI Appendix*, Fig. S7.

### Manual Methane Flux Measurements.

Pine shoot methane exchange was quantified in static chamber systems, i.e., shoots were enclosed in a gas-tight chamber and air was recirculated between the enclosure and a trace gas analyzer. Two different measurement systems were used, to which we refer as “manual” and “automated.” Measurements under field settings and in the greenhouse yard were conducted with the manual chamber system described in ref. 5. Approximately 30 cm long shoots were placed in cylindrical frames (volume 5.2 L) that were covered with UV-transparent fluoroethylene polymer (FEP) film during measurements. The opening where the branch passed into the chamber was tightened with a pressure-sensitive adhesive (Blu Tack; Bostik SA, Colombes, France). Measurements were conducted at lateral shoots of the upper crown at Hyytiälä and at apical shoots in the greenhouse yard. The enclosure was then connected to a Los Gatos Research UGGA trace gas analyzer through PTFE tubing. The method detection limit for a single manual closure, defined as three times SD of the apparent flux observed in empty chamber measurements, was 0.389 nmol CH_4_ g^−1^ dw h^−1^.

### Automated Methane Flux Measurements.

The automatic system used for measurements in a greenhouse compartment was described recently (33). Briefly, the system operates seven custom-built shoots chambers with a volume of 1.15 L (total volume for one measurement loop 1.6 L) each. Six of the chambers were on the lateral shoot of a sapling while the seventh chamber was left empty as a control. Measurements were conducted over 33 d from May 7 to June 9, 2020.

The chambers are set up such that they can be operated both in static chamber mode, i.e., circulating air between the chamber and a gas analyzer to quantify trace gas fluxes, and in a dynamic mode, i.e., the chamber being continuously flushed with air to quantify CO_2_ and water fluxes. Each shoot chamber was cooled to within 2 °C of the ambient temperature using a Peltier element throughout the measurement campaign. Methane exchange was measured in the static mode by monitoring its concentration by cavity ring-down laser spectroscopy (Picarro G2301), a method previously shown to have low interference from VOCs (31, 33). During these measurements, transpired water was removed from the chamber air with a membrane drier tube (Nafion) and a fixed volume of CO_2_ corresponding to ca. 700 ppm was added whenever the CO_2_ concentrations fell below 400 ppm during these static mode closures. Each closure lasted ca. 20 min, such that each of the seven chambers was measured once every 3 h. CO_2_ and water exchange were measured in the dynamic mode by mid infrared spectroscopy (Licor LI-850). Data processing was conducted as described previously (33). In addition, we filtered outlying values based on Rosner’s test applied to the residuals of a model using chamber and measurement time as predictors. The method detection limit of the automated chamber measurements was 0.233 nmol g^−1^ dw h^−1^ for a single 20-min closure and 0.041 nmol g^−1^ d.w. h^−1^ for the average flux from one shoot during a point in the diurnal cycle (*n* = 31 to 33). More details on detection limit calculations are provided in *SI Appendix*, *Text*.

### Foliar Dry Mass.

Shoot dry mass was determined after drying at 65 °C for 72 h.

### Flux Calculations.

Flux calculations were conducted as described in detail previously (12, 33). In the greenhouse experiment, mixing ratios of methane were first corrected for dilution by CO_2_ injections as described in ref. 33. Methane fluxes in all experiments and CO_2_ fluxes in the garden experiment were calculated based on the static chamber principle, i.e., based on the change of the analyte’s mixing ratio over time in an enclosed air volume. This was done according to Eq. **1**,

[1] $$\begin{array}{c} F=\frac{\mathrm{dC}}{\mathrm{dt}}\cdot \frac{V}{m}\cdot \frac{p}{R\cdot T}, \end{array}$$

where *F* stands for the flux in mol g^−1^ dw s^−1^, dC/dt stands for the change in mixing ratio in dry air over time in s^−1^. dC/dt was derived from measurements using linear (methane) or exponential (CO_2_) fits, V for the chamber volume in L, m for the shoot foliar dry mass in g, p for the atmospheric pressure (assumed 101325 Pa), R for the ideal gas constant (831 L Pa K^−1^ mol^−1^), and T for the temperature in K. To filter for outliers, we calculated the difference between each flux and the average of all measurements conducted at the same shoot and time-of-day. We then combined these residuals (*n* = 1,602) and applied Rosner’s test, which identified 38 outliers (2.4 % of measurements) that were removed from the dataset.

In the greenhouse experiment, CO_2_ and water fluxes were calculated based on dynamic chamber principle (Eq. **2**). Fluxes (F) were calculated as the difference in the analyte’s mixing ratio in ingoing ($C_{\mathrm{in}}$) and outgoing ($C_{\mathrm{out}}$) air multiplied by the flow rate (0.85 L min^−1^) and divided by the foliar dry weight *m*.

[2] $$\begin{array}{c} F=\frac{\mathrm{flowrate}}{m}\cdot \left(C_{\mathrm{out}}-C_{\mathrm{in}}\right)\cdot \frac{p}{R\cdot T}. \end{array}$$

Vapor pressure deficit (VPD) and stomatal conductance (g_s_) were calculated according to the equations given in ref. 33.

### Ecosystem Flux Measurements.

Above-canopy eddy covariance (EC) measurements were conducted at 22.5-m height from June 15th to December 31st 2017. The EC system included an ultrasonic anemometer (Solent Research HS1199, Gill Instruments, Lymington, UK) to measure three wind velocity components at 10 Hz frequency and a closed-path fast response gas analyzer (G2311-f, Picarro Inc.), which measured methane, CO2, and water vapor mole fractions at 10 Hz. The intake line was 24 m long, and the flow rate was approximately 10 L min^−1^. The EC fluxes were calculated by using the EddyUH software (49) as 30-min block-averaged covariances between the methane dry mole fraction and the vertical wind velocity following ICOS recommendations given in refs. 50 and 51. In order to eliminate outliers, the 10 Hz raw data were despiked according to standard methods (52), and two-step coordinate rotation was used to rotate the coordinate frame of wind velocity components. Assuming scalar similarity, the time lag between the vertical wind speed and methane concentration measurement was determined from the maximum cross-covariance of CO_2_ with vertical wind speed. Fluxes were corrected for high and low frequency spectral losses according to ref. 53, and the EC system response time of CO_2_ was also used for methane spectral corrections.

### Partitioning of Ecosystem Methane Fluxes.

Eddy covariance derived fluxes ($F_{\mathrm{eco}}$) were partitioned into a light dependent ($F_{\mathrm{ld}}$) and a light independent ($F_{\mathrm{li}}$) component using the model $F_{\mathrm{eco}}=F_{\mathrm{ld}}+F_{\mathrm{li}}$. where is $F_{\mathrm{li}}=a\cdot T$ and $F_{\mathrm{ld}}=b\cdot PAR$, with T being the air temperature at 16.8 m height, PAR the photosynthetic radiation, and a and b fitted parameters. In practice, we determined the temperature dependence of $F_{\mathrm{li}}$ (a) based on dark periods (PAR < 50 μmol m^−2^ s^−1^), extrapolated $F_{\mathrm{li}}$ to light periods, and subtracted $F_{\mathrm{li}}$ from $F_{\mathrm{eco}}$. We then binned the residual based on PAR classes (0.1 mmol m^−2^ s^−1^ wide) and calculated the mean and SE for each bin. We then determined b using a weighted linear regression between bin center (PAR value) and the mean residual methane flux observed for the bin (Fig. 4*B*). This regression was weighted by the inverse SE of each bin. The light-dependent component was then calculated as $F_{\mathrm{ld}}=PAR\cdot b$. To scale $F_{\mathrm{ld}}$ to foliar dry weight, we assumed a leaf area index of 4.5 m_2_ m^−2^ (48) and a specific leaf area of 4.38 m_2_ kg^−1^ (54).

### Internal Methane Concentrations in Scots Pine Stems.

Measurements were conducted at 14 mature Scots pine trees in the footprint of the flux tower in August 2022 as part of an international collaborative study (MethaneTraits). We followed the MethaneTraits protocol: Each tree was cored to the center using a 5-mm increment borer. After removing the wood sample, the corer was extracted halfway and the opening of the corer was sealed. After allowing the air in the borer hole to equilibrate for 5 min, a sample of 15 mL was collected into a gas-tight vial. Methane concentrations were measured using an Agilent 7890B gas chromatograph equipped with a flame ionization detection as described previously (55).

### Targeted Metagenomics.

To reveal the potential effect of microbial CH_4_ production to the measured CH_4_ fluxes, we analyzed presence of the functional gene mcrA (coding for methyl coenzyme M reductase in methanogenic archaea) via targeted metagenomics. Pine shoots were sampled in the greenhouse yard in connection to the flux measurements done on May 8 2019. Samples were stored in −80 °C until DNA was extracted with the Nucleospin Plant II kit (Macherey-Nagel, Düren, Germany). DNA was extracted separately from the buds, needles, and woody stem parts of the shoots (*n* = 3 in each group), and its quantity and quality were checked with the Qubit fluorometer and the Nanodrop One spectrophotometer (both Thermo Fisher Scientific). Targeted sequencing was conducted by Daicel Arbor Biosciences (US) as in ref. 29. Data were analyzed as described in ref. 29. No mcrA homologs were detected via HMMER screening in any of the samples (*SI Appendix*, Table S3). As positive controls for the targeted sequencing protocol, we used a) parallel detection of nifH genes in the pine same samples via nifH-specific probes (*SI Appendix*, Table S3) and b) detection of mcrA genes in Salix lapponum and Menyanthes trifoliata using the same protocol reported separately (56). Raw sequence data has been deposited to the SRA database with project number PRJNA1024526.

### Data Analysis.

All data analysis was conducted using the statistical programming language R version 4.2.1. *T*-tests were used for pairwise comparison between chambers with and without shoots and between daytime and nighttime measurements (Fig. 2). In addition, we tested for differences between daytime and nighttime fluxes used mixed effects models with Shoot ID as a random effect (r packages *lme4* v. 1.1-32 and *lmerTest* 3.1-3). To demonstrate differences throughout the diurnal cycle, we grouped data into 3-h time-of-day groups and again used mixed effects model with Shoot ID as a random variable. Differences between time-of-day groups were tested using estimated marginal means (*emmeans* package version 1.8.8). The diurnal and seasonal patterns plotted in Fig. 3 were fit using either a general additive model (function gam() in *mgcv* package) with shoot ID as a random variable and customized nodes positions to avoid overfitting during light on-off transitions (Fig. 3), or a simple local regression model (loess() in *stats*; Fig. 4).

## Supplementary Material

Appendix 01 (PDF)

## Data Availability

Raw flux data have been deposited in Zenodo (https://doi.org/10.5281/zenodo.8027450) (57).
