# Supplementary material for: Radiation and temperature drive diurnal variation of aerobic methane emissions from Scots pine canopy
Source: Proc Natl Acad Sci U S A. 2023 Dec 21;120(52):e2308516120. doi: 10.1073/pnas.2308516120 (PMC10756279; doi:10.1073/pnas.2308516120)
Supplement: Supplementary file 1 — Appendix 01 (PDF) [file pnas.2308516120.sapp.pdf]

Supporting Information for

Radiation and temperature drive diurnal variation of aerobic methane emissions from Scots pine canopy

Lukas Kohl, Salla A. M. Tenhovirta , Markku Koskinen, Anuliina Putkinen, Henri M.P. Siljanen, Iikka Haikarainen, Tatu Polvinen, Marjo Patama, Luca Galeotti, Ivan Mammarella, Thomas Matthew Robson, Bartosz Adamczyk, Mari Pihlatie

Lukas Kohl.
E-mail: lukas.kohl@uef.fi

This PDF file includes:

Supporting text
Figs. S1 to S8
Tables S1 to S4
SI References

Supporting Information Text

Supporting Methods - Detection Limit. We calculated the following detection limits primarily as an expression of the measurement precision primarily for method inter-comparison and as a determine if data points should not be interpreted individually because each datapoint by itself may have analytical noise. Note that datapoints below the detection limit should not be removed before statistical analysis (1). We further estimate the detection limit for the average of repeated measurements, which we provide primarily as a parameter describing the analytical sensitivity of our method. We do not rely on the so-calculated detection limits to determine the presence or absence of methane exchange between shoot and atmosphere. Rather, we use statistical tests (comparison between chambers with and without shoots) to evidence methane fluxes (see below) and use error propagation to estimate confidence intervals that include uncertainties derived from the uncertainties in both measurements and blanks.

Instrument Detection Limit. We calculated the instrument detection limit, that is, the minumum detectable flux based on analyzer precision alone, according to (2, 3), as stated in eq. 1

$$MDF = \frac{A_A}{t_c \cdot \sqrt{t_c/p_s}} \cdot \frac{V \cdot p}{w \cdot R \cdot T} \quad [1]$$

where MDF stands for the minimum detectable flux in nmol g^{-1} dw h^{-1}, t_c for the closure time in hours (7 minutes in forest and garden measurements, 20 minutes in greenhouse measurements), V for the chamber volume (5.2L in forest and garden measurements, 1.6 L in greenhouse measurements), m for the shoot foliar dry mass in g, p for the atmospheric pressure (assumed 101325 Pa), R for the ideal gas constant (831 L Pa K^{-1} mol^{-1}), and T for the air temperature in the chamber (assumed 298 K). A_A stands for the precision (3SD) of the analyser in ppb when integrated over time p_s. We applied the analyser precision for specified by the manufacturer for either 5 minute (Picarro G2301, 1SD <0.22 ppb) or 100 sec integration (LGR UGGA, 1SD <0.25 ppb). The so-calculated analytical detection limits were 0.028 nmol g^{-1} dw h^{-1} in the forest and garden measurements and 0.013 nmol g^{-1} dw h^{-1} in the greenhouse measurements. In other words, the fluxes detected in our study were well above the minimal fluxes theoretically measurable with both analysers.

Method detection limit. The method detection limit includes measurement uncertainties resulting from analyser noise, but also uncertainties from other sources like chamber leakage (4). We calculated these method detection limits based on the best available blank measurement, that is measurements conducted with empty chambers placed in close proximity of the actual shoot chambers. The detection limits of these measurements were calculated as 3 standard deviations of the apparent flux observed in empty chambers. The minimum detectable fluxes based on the method detection limits were 0.387 nmol g^{-1} dw h^{-1} in the forest and garden measurements and 0.233 nmol g^{-1} dw h^{-1} in the greenhouse measurements.

Repeated measurements on individual shoots. As measurement precision increases with number of measured replicates, the detection limit of decreases. In the case of normally distributed and independent measurement uncertainty ('white noise'), the detection limit decreases with the square root of the averaged measurements (5, 6). These assumptions are typically proved with so-called Allen Variance plots, which are provided for manual and automated measurements in Fig. S8. These plots demonstrate that the measurement precision indeed improves as expected with increasing replicate numbers. We therefore follow calculate the detection limit for repeated measurements on one shoot according to (6).

We aggregated, for example fluxes in three-hour time-of-day groups for statistical comparison. In the manual garden measurements, each of four shoots was measured on average 3.6 times in a three hour interval (combining the two measurement days, range 2-5). The theoretical detection limit for the average in a block was therefore 0.204 nmol g^{-1} dw h^{-1}. In the greenhouse experiment, an average 32.6 measurements per shoot and three-hour interval (once per day over 33 days, range 31-33) were available, and the theoretical detection limit for the average of a three-hour interval was 0.041 nmol g^{-1} dw h^{-1}. We refer to these detection limits because they were calculated based on statistical assumptions. We did not use these calculated detection limits to determine the presence or absence of methane fluxes between shoot and atmosphere, but rather made such inference based on statistical tests.

Repeated measurements on distinct shoots. To account for repeated measurements on distinct shoots, we used mixed effects models which account for repeated measurements on individual shoots and random variation between distinct shoots (see results below). The average uncertainties (2 SE) of the estimated flux for a three-hour interval (fixed effects) were 0.102 nmol g^{-1} dw h^{-1} and 0.029 nmol g^{-1} dw h^{-1} in the manual and automatic measurements, respectively. These uncertainties can serve as an estimate of the minimal detectable average flux during a three-hour time-of-day interval after merging the data measured on distinct shoots.

We calculated the following detection limits primarily as an expression of the measurement precision primarily for method inter-comparison and as a determine if data points should not be interpreted individually because each datapoint by itself may have analytical noise. Note that datapoints below the detection limit should not be removed before statistical analysis (1). We further estimate the detection limit for the average of repeated measurements, which we provide primarily as a parameter describing the analytical sensitivity of our method. We do not rely on the so-calculated detection limits to determine the presence or absence of methane exchange between shoot and atmosphere. Rather, we use statistical tests (comparison between chambers with and without shoots) to evidence methane fluxes (see below) and use error propagation to estimate confidence intervals that include uncertainties derived from the uncertainties in both measurements and blanks.

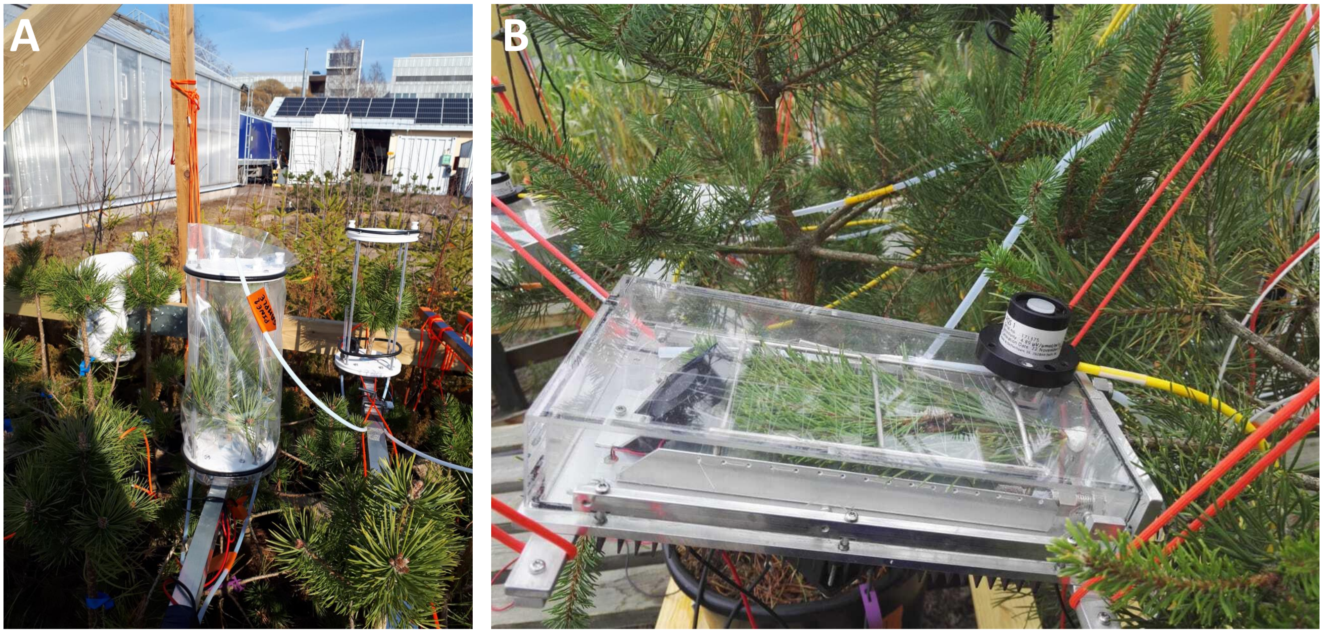

Fig. S1. Images of the shoot chambers employed in the Forest and Garden measurements (A) and the Greenhouse experiment (B).

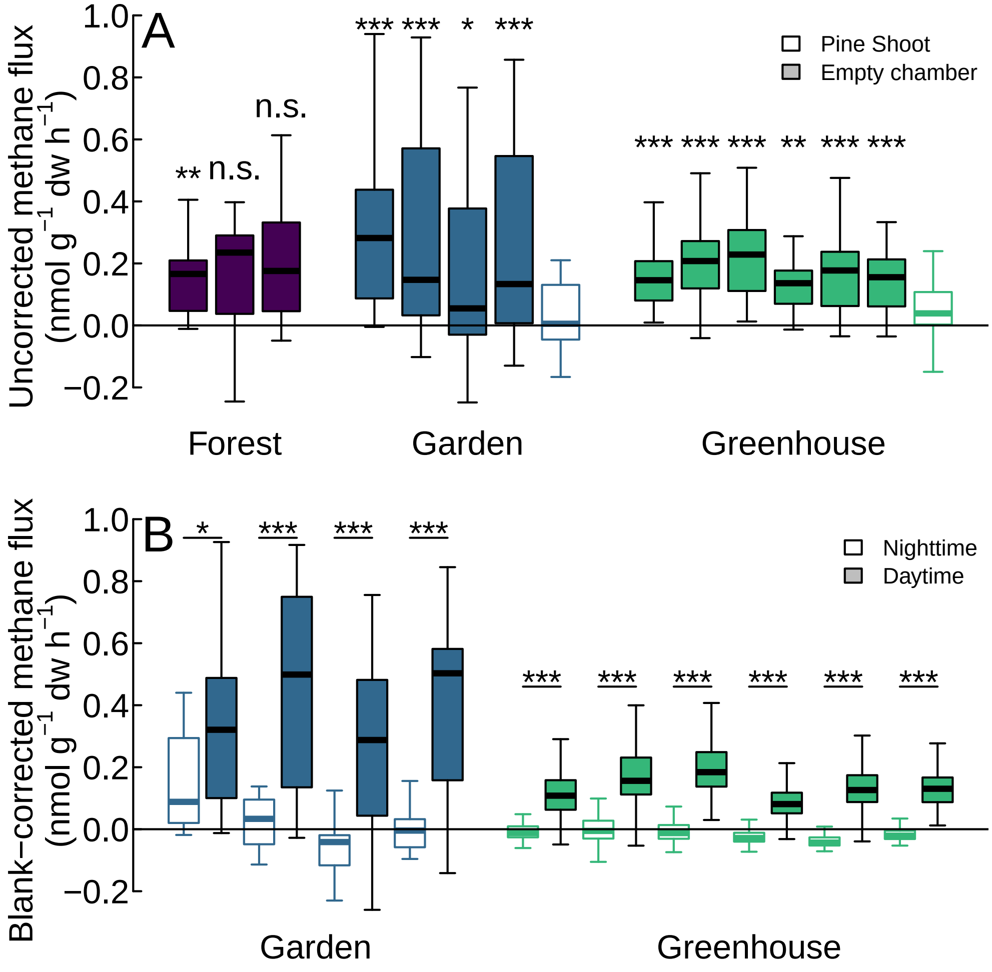

Fig. S2. Shoot-wise comparison of the measured methane fluxes from Scots pine shoots to empty chamber controls (A) and between daytime and nighttime fluxes from each shoot. Asterisks indicate significant differences between shoot chamber and empty chamber (A, t-test) or between daytime and nighttime flux (B, t-test). *, p <0.05; **, p <0.01; ***, p <0.001; n.a., not significant.

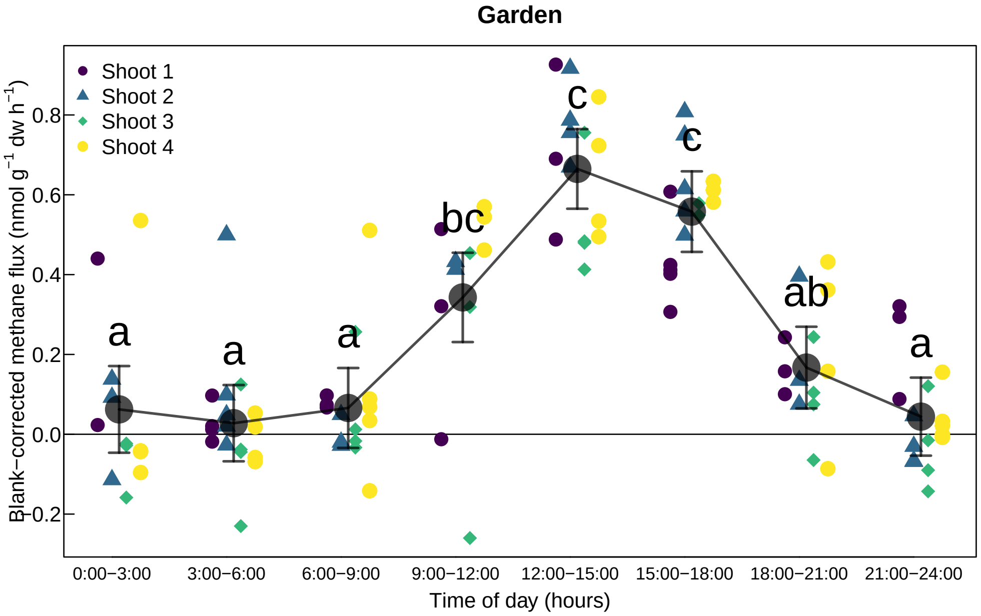

Fig. S3. Diurnal variability of shoot methane fluxes in the garden campaign. Fluxes were stratified into three-hour time-of-day groups. Differences between groups were assessed using two mixed effect models with time-of-day group as a fixed effect and shoot ID as a random slope. Letters indicate significant differences between time groups. Four time groups (9:00 to 21:00) had average fluxes significantly different from zero.

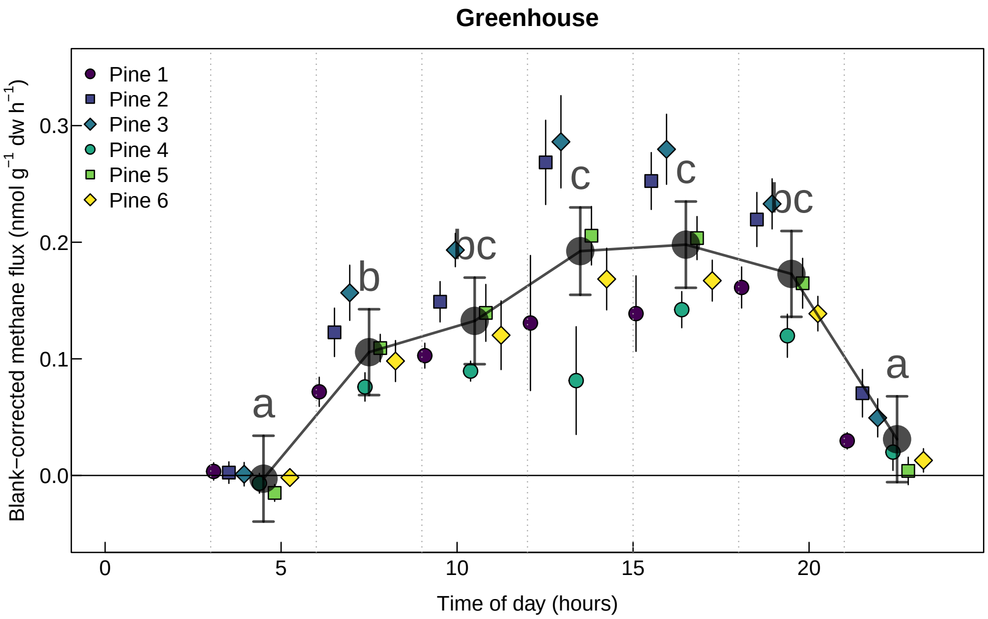

Fig. S4. Diurnal variability of shoot methane fluxes in the greenhouse campaign. Fluxes were stratified into three-hour time-of-day groups. Differences between groups were assessed using two mixed effect models with time-of-day group as a fixed effect and shoot ID as a random slope. Letters indicate significant differences between time groups.Five time-of-day groups (6:00 to 21:00) had average fluxes significantly different from zero.

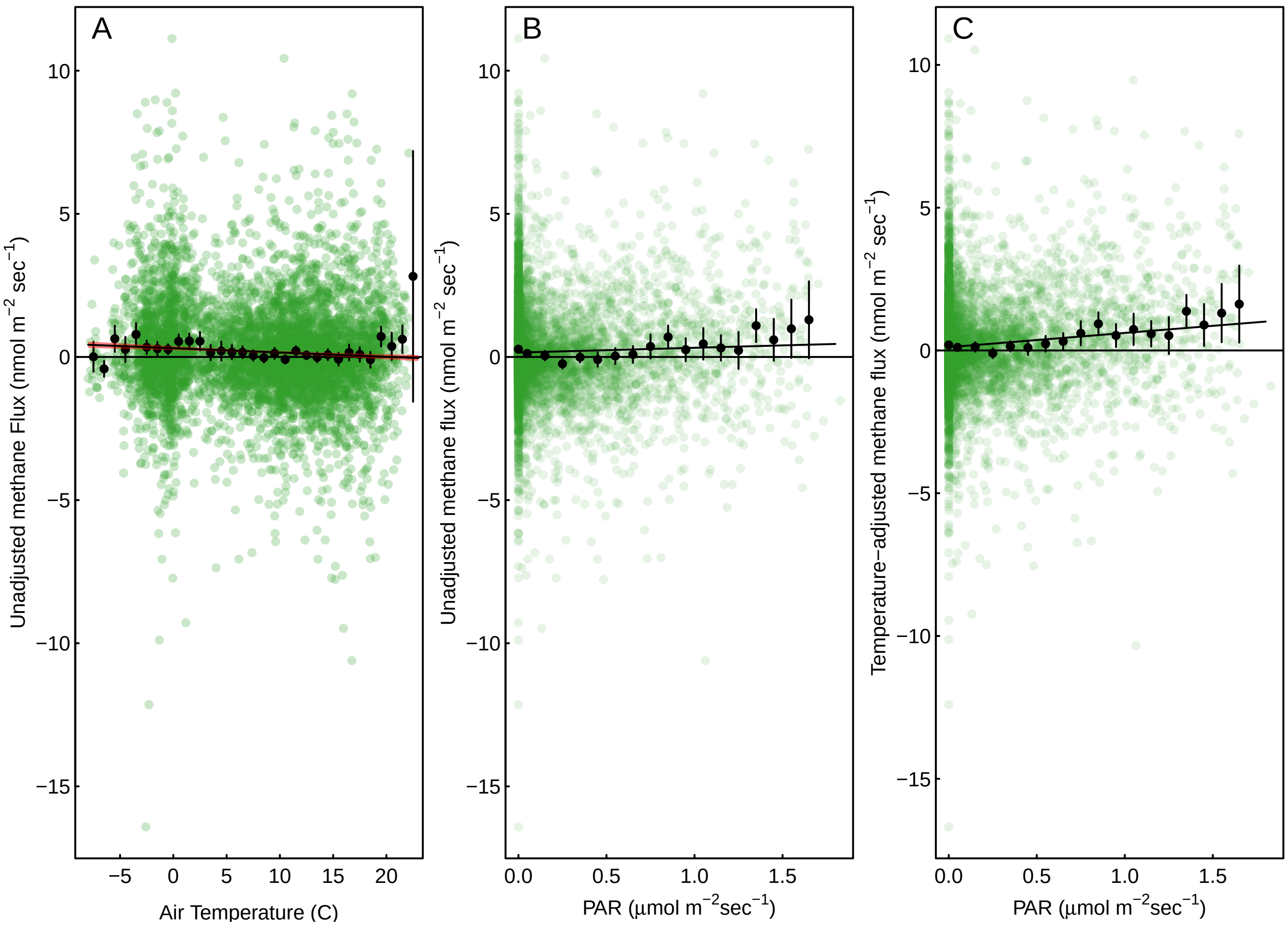

Fig. S5. Relation of ecosystem-level methane fluxes with ambient temperature (**A**) and photosynthesis-active radiation (**B**). Further, the relationship of methane fluxes adjusted for temperature with PAR **C**). Raw fluxes are plotted in the background with black lines indicate linear regressions and shaded red areas representing 95% confidence intervals. Black symbols and error bars represent means ±95% confidence intervals of methane fluxes binned by temperature (1 °C) or PAR (0.1 mmol m^{-2} sec^{-1}).

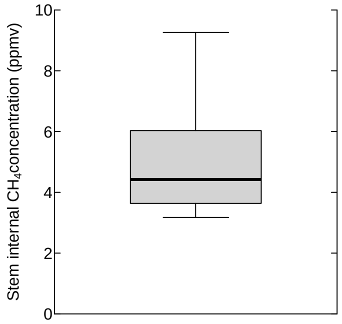

Fig. S6. Internal methane concentration in Scots pine stems in the Hyytiälä research forest. Raw data is provided in Table S3

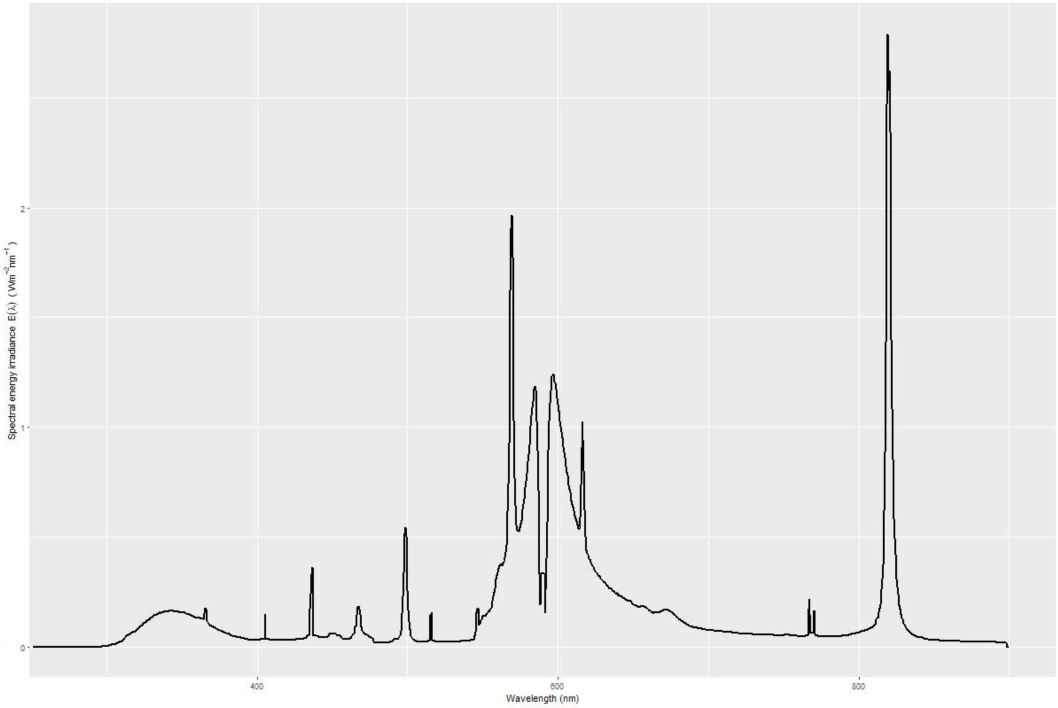

Fig. S7. Spectral irradiance received at a Scots pine shoot in the greenhouse experiment. The spectrum was measured under the cover of a shoot chamber in February 2020.

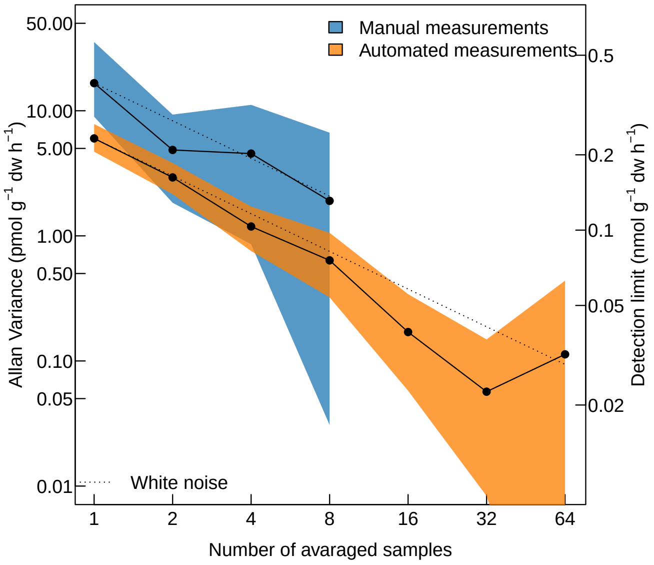

Fig. S8. Allen Variance plots and derived detection limits for manual and automated measurements. The detection limit was defined as three times the Allan deviation.

Table S1. Literature review of shoot methane emissions measured in field and laboratory settings. Measurements on detached leaves were not included in the list.

Species	Season	Location	Chamber method	Analyser	CH4 flux (nmol g^{-1} dw h^{-1})	CH4 flux reported?	source
Field measurements							
Japanese cypress	full year	Kiryu Exp. Watershed, Japan	static	LGR FMA-100	0±16[b,c]	no	(7)
Various	Fall	Sweden	static	LGR ICOS	-3.5[e]	yes	(8)
various	Summer	Haliburton Forest, Canada	static	LGR ICOS	-9.7±2.2[b,d]	yes	(9)
Scots pine	Summer	Hyytiälä Res. Forest, Finland	static	GC-FID	0.018[e]	yes	(10)
Betula pubescens	Spring	Hyytiälä Res. Forest, Finland	static	GC-FID	0.14-1.4[e]	N/A	(11)
Piceia abies	Spring	Hyytiälä Res. Forest, Finland	static	GC-FID	0.25-0.275[e]	N/A	(11)
Scots pine	Summer	Hyytiälä Res. Forest, Finland	static	LGR UGGA	0.167±0.079	yes	This study
Scots pine	Spring	Helsinki, Finland	static	LGR UGGA	0.043±0.010	yes	This study
Laboratory measurements							
Maize, wheat, sweet vernal grass			static	GC-IRMS	0.75-23	yes	(12)
Various, (^{13}C-labelled plants)			dynamic	PAS	–0.6 to 2.6	no	(13)
Tobacco			static	GC-FID	0.810	yes	(14)
Maize, tobacco			dynamic	FID	0±2-3 [h]	no	(15)
Pea			static	GC-FID	18.25 (mean)	yes	(16)
Grey poplar, (^{13}C-labelled plants)			static	GC-FID	0.01-0.04	yes	(17)
Maize			static	GC-FID	-0.5±0.24	no	(18)

[a] Positive value indicate methane emissions, negative values m,ethane uptake. Measurements below the detection limit (LOD) are stated as 0±95% confidence interval. [b] Mean ±95 % confidence interval. [c] Converted from original source based on specific leaf area (SLA) = 4.64 m^2 kg^{-1} (50). [d] Median. [e] Converted from original source based on SLA=5 m^2 kg^{-1}. [e] Medians of measurements on two shoots. [g] converted source based on SLA=4.38 m^2 kg^{-1} (36) [h] 95% confidence interval for two plants

Table S2. Internal methane concentration in Scots pine stems.

Tree ID	Diameter at breast height (cm)	CH_4 concentration (ppmv)
Ps1	27.6	6.51
Ps2	28.1	4.13
Ps3	22.3	3.18
Ps4	23.9	3.17
Ps5	23.6	4.73
Ps6	21.8	3.64
Ps7	22.2	3.60
Ps8	21.3	6.52
Ps9	21.2	6.03
Ps11	24.0	4.10
Ps12	27.1	9.26
Ps13	25.6	3.99
Ps14	24.6	4.72
Ps15	21.9	5.74

Table S3. Targeted metagenomics: Total and functional gene fasta reads. Total reads indicate the number of reads produced by sequencing, mcrA (methanogens) and nifH (nitrogen fixers, as positive control) reads indicate the number of reads assigned to these genese by HMMER-searches with inclusion threshold of Inc-E 0.0001, verified nifH reads indicate the number of reads that were confirmed by alignment with known nifH sequences in the reference gene database with using MAFFT (19).

Sample	file name	total reads	mcrA reads (HMMER)	nifH reads (HMMER)	verified nifH reads (MAFFT)
Scots pine sapling 1, bud	16_R1R2.fasta	8 227012	0	3561	0
Scots pine sapling 1, needle	17_R1R2.fasta	6 959858	0	15394	0
Scots pine sapling 1, stem	18_R1R2.fasta	7 895262	0	6007	13
Scots pine sapling 2, bud	19_R1R2.fasta	7 713852	0	3665	0
Scots pine sapling 2, needle	20_R1R2.fasta	5 916318	0	8848	0
Scots pine sapling 2, stem	21_R1R2.fasta	16 126162	0	14605	1211
Scots pine sapling 3, bud	22_R1R2.fasta	13 735434	0	4367	14
Scots pine sapling 3, needle	23_R1R2.fasta	8 526088	0	14085	158
Scots pine sapling 3, stem	24_R1R2.fasta	20 266956	0	14978	295

References

1. Analytical Methods Committee, Recommendations for the definition, estimation and use of the detection limit. *The Analyst* **112**, 199–204 (1987).
2. JR Christiansen, J Outhwaite, SM Smukler, Comparison of CO2, CH4 and N2O soil-atmosphere exchange measured in static chambers with cavity ring-down spectroscopy and gas chromatography. *Agric. For. Meteorol.* **211-212**, 48–57 (2015).
3. N Nickerson, *Evaluating gas emission measurements using Minimum Detectable Flux (MDF).* (Eosense Inc., Dartmouth, Nova Scotia, Canada) No. March, p. 6 (2016).
4. L Kohl, et al., An automated system for trace gas flux measurements from plant foliage and other plant compartments. *Atmospheric Meas. Tech.* **14**, 4445–4460 (2021).
5. P Werle, R Mücke, F Slemr, The limits of signal averaging in atmospheric trace-gas monitoring by tunable diode-laser absorption spectroscopy (TDLAS). *Appl. Phys. B Photophysics Laser Chem.* **57**, 131–139 (1993).
6. Eurachem, *Earachem Guide: The Fitness for Purpose of Analytical Methods - A Laboratory Guide to Method Validation and Related Topics.* eds. B Magnusson, U Örnemark. Vol. 56, 2nd edition, p. 544 (2014).
7. K Takahashi, Y Kosugi, A Kanazawa, A Sakabe, Automated closed-chamber measurements of methane fluxes from intact leaves and trunk of Japanese cypress. *Atmospheric Environ.* **51**, 329–332 (2012).
8. E Sundqvist, P Crill, M Mlder, P Vestin, A Lindroth, Atmospheric methane removal by boreal plants. *Geophys. Res. Lett.* **39**, L21806–L21806 (2012).
9. AS Gorgolewski, JP Caspersen, J Vantellingen, SC Thomas, Tree Foliage is a Methane Sink in Upland Temperate Forests. *Ecosystems* **26**, 174–186 (2023).
10. K Machacova, et al., Pinus sylvestris as a missing source of nitrous oxide and methane in boreal forest. *Sci. Reports* **6**, 1–8 (2016).
11. E Vainio, et al., Soil-tree-atmosphere CH4 flux dynamics of boreal birch and spruce trees during spring leaf-out. *Plant Soil* **478**, 391–407 (2022).
12. F Keppler, JTG Hamilton, M Braß, T Röckmann, Methane emissions from terrestrial plants under aerobic conditions. *Nature* **439**, 187–191 (2006).
13. TA Dueck, et al., No evidence for substantial aerobic methane emission by terrestrial plants: a 13C-labelling approach. *New Phytol.* **175**, 29–35 (2007).
14. AR McLeod, et al., Ultraviolet radiation drives methane emissions from terrestrial plant pectins. *New Phytol.* **180**, 124–132 (2008).
15. DJ Beerling, T Gardiner, G Leggett, A McLeod, P Quick, Missing methane emissions from leaves of terrestrial plants. *Glob. Chang. Biol.* **14**, 1821–1826 (2008).
16. MM Qaderi, DM Reid, Methane emissions from six crop species exposed to three components of global climate change: Temperature, ultraviolet-B radiation and water stress. *Physiol. Plantarum* **137**, 139–147 (2009).
17. N Brüggemann, et al., Nonmicrobial aerobic methane emission from poplar shoot cultures under low-light conditions. *New Phytol.* **182**, 912–918 (2009).
18. MUF Kirschbaum, A Walcroft, No detectable aerobic methane efflux from plant material, nor from adsorption/desorption processes. *Biogeosciences* **5**, 1551–1558 (2008).
19. A Putkinen, et al., New insight to the role of microbes in the methane exchange in trees: evidence from metagenomic sequencing. *New Phytol.* **231**, 524–536 (2021).
20. MUF Kirschbaum, et al., A comment on the quantitative significance of aerobic methane release by plants. *Funct. Plant Biol.* **33**, 521 (2006).
21. AJ Parsons, PCD Newton, H Clark, FM Kelliher, Scaling methane emissions from vegetation. *Trends Ecol. Evol.* **21**, 423–424 (2006).
22. CL Butenhoff, MAK Khalil, Global methane emissions from terrestrial plants. *Environ. Sci. Technol.* **41**, 4032–4037 (2007).
23. AA Bloom, et al., Global methane emission estimates from ultraviolet irradiation of terrestrial plant foliage. *New Phytol.* **187**, 417–425 (2010).

Table S4. Literature review of global estimates of aerobic methane emissions.

Upscaling based on	Emission factor in source (nmol g^{-1} dw h^{-1})	Global flux in source (Tg CH_4 yr^{-1})	Global flux using emission factor in this study[a] (Tg CH_4 yr^{-1})	Source (Tg CH_4 yr^{-1})
Net Primary Production	23.4 (12.4-37.4)	149 (62-236)	0.99	(12)
Leaf Biomass	23.4 (12.4-37.4)	36.4 (15.1-60.3)	0.24	(20)
Photosynthesis	23.4	9.6	0.06	(20)
Leaf Biomass	23.4	42	0.28	(21)
Leaf Area	23.4	14-60	0.09-0.40	(22)
Foliage Biomass	23.4	8-34	0.05-0.23	(22)
Pectin / UV measurements		0.2-1.0		(23)

[a] Average flux observed in forest measurements (0.121 nmol g^{-1} dw h^{-1}).
